# Insights from the sequence similarity of Zika virus proteins with the Human nerve proteins

**DOI:** 10.6026/97320630014194

**Published:** 2018-05-31

**Authors:** Prasanna Marsakatla, Sujai Suneetha, Joshua Lee, Paari Dominic Swaminathan, Logeshwaran Vasudevan, Rachael Supriya, Lavanya Moses Suneetha

**Affiliations:** 1 CODEWEL Nireekshana ACET, Narayanaguda, Hyderabad -500029, Telangana, India; 2 York University, Department of Science, 4700 Keele St, Toronto, ON M3J 1P3, Canada; 3 LSU Health Sciences Center, Center for Cardiovascular Diseases and Sciences Shreveport, LA, USA; 4 Presidency College, Department of Zoology, Chennai-05, India

**Keywords:** Zika V, Nerve tissue proteins, Neuropathogenesis, Bioinformatics, BLAST, Neuromodulin, Nestin, Bombesin, Galanin, Calcium-binding protein

## Abstract

Massive peptide sharing between the Zika virus polyprotein and host tissue proteins could elicit significant host-pathogen interactions and cross-reactions leading to autoimmune diseases. This study found similarities in the Zika V proteins and human nerve tissue proteins. 63 human nerve proteins were screened for similarities with the Zika V of which Neuromodulin, Nestin, Galanin, Bombesin, Calcium-binding protein were found to have similarities to the Zika V poly protein C at different sequence regions. These sequence similarities could be significant in regulating pathogenic interactions/autoimmunity, as Polyprotein C is known to be a virulent factor.

## Background

The Zika Virus (Zika V), is an emerging infectious disease agent causing human birth defects. It has created a global alarm and was declared a public health emergency of international concern by the World Health Organization (WHO) [1]. The Zika V proteome has been sequenced. Its role in infection, inflammation and pathogenesis of the human nerve are being extensively investigated. Recent studies have revealed that the Zika V shows preferential infection to neural progenitor cells of a mouse brain and when it infects the neural stem cells and immature neurons, results in alterations in the gene expression of cell cycle related proteins inducing neural-cell death and reduced production of new neurons. The decreased proliferation of the neural cells could cause fetal microcephaly in infected pregnant women [2, 3]. Selective permeability permitting the Zika V to cross the foetal blood-brain barrier has also been indicated [4, 5]. Acute infection in these patients leads to a polyfunctional T-cell activation along with increased response of its respective cytokines (IL-1β, IL-2, IL-4, IL-6, IL-9, IL-13, IL-17, IFN-γ) and growth factor responses [RANTES, macrophage inflammatory protein 1α(MIP1α) and vascular endothelial growth factor response (VEGF)] [6]. Research on the various cells targeted by the Zika V revealed the engagement of several host adhesion factors (DC-SIGN, AXL, Tyro3, and TIM-1) facilitating the entry of the Zika V into different tissue cells [7]. Cell culture experiments of the Zika V infection expressed transcription of Toll-like receptor 3 (TLR3), retinoic acid-inducible gene 1 (RIG-I) and melanoma differentiation-associated protein 5 (MDA5) [7].

The molecular mechanism of the pathogen's interaction with the host and its use in drug discovery is at an experimental stage [8, 9]. Though the pathogenic pathway of infectious agents across various host tissues is distinctive and often undefined, many of these processes can be attributed to a role of molecular mimicry between pathogen and its corresponding host tissue proteins [10, 11]. A study identified the sequence and structural similarities between Mycobacterium leprae and the immunoglobulin regions of Myelin P0, which could be the contributing factor to autoimmunity to myelin P0 amongst Leprosy patients with peripheral nerve damage [12, 13, 14, 15]. The sequence and structural similarities between the Zika V Virulant Factor and host nerve peptides could directly or indirectly impact the pathogenesis of the disease [16]. There is insilco evidence revealing massive peptide sharing between the Zika V protein and host tissue proteins causing cross-reactions inducing autoimmunity. Recent research demonstrated reveals the expression of unique transcriptomic signatures in Zika V infected human neural stem cells [17, 18].

However, to the best of our knowledge there is no report on major human nerve tissue protein similarities to the Zika V proteins. The hypothesis is that sequence and structural similarities (mimics) that exist in the host nerve and pathogen proteins include significant host-pathogen cross reactions - i.e. in receptor binding, steric hindrance, signalling/transmission, metabolic alteration, inflammation and auto-antibody production, which could ultimately lead to aberrant development of neurons and neuropathy [19, 20]. To assess whether such sequence similarities /molecular mimics occurred between the Zika V and the human host, we compared the peptide sequence of 63 proteins expressed in the human nerve tissue with that of the peptide sequence of the Zika V polyprotein with the use of bioinformatic tools.

## Methodology

64 human nerve proteins were selected to be BLAST (Basic Local Alignment Search Tool; Version 2.2.28; e-value ≤ 0.01) against the Zika V proteome (Tax ID 64320). The peptide sequence similarities between the host and counterpart proteins were identified in the PSI-Blast (BLASTP 2.6.0+) by their aa/nucleotide positions [21].

### Selection of nerve proteins

64 proteins (Table 1) that were enriched and enhanced in the nervous tissue demonstrated by immunohisochemistry were extracted from the Human Protein Atlas Database (www.proteinatlas.org). FASTA formats for each of the above proteins were retrieved from NCBI PubMed and were saved in a Microsoft notepad to be BLAST against the Zika V proteome (Tax ID 64320). The output of the BLAST identified significant peptide sequence similarities between the human protein and its pathogen counterpart. Similarities identified in the peptide sequence region of Neuromodulin was superimposed on the of the viral protein Cryo-em structure of the immature Zika V structure (PDB ID: 5U4W_A) using the Visual Molecular Dynamics 1.9.1 (VMD) modelling software.

## Results

Nestin, Bombesin, Galanin, Calcium Binding Protein and Neuromodulin were found to mimic the Cryoem- protein and various other peptide regions of the polyprotein C in the Zika V proteome.

### Sequence similarity of Neuromodulin peptide on ZIKV polyprotein C

**Figure 1:**
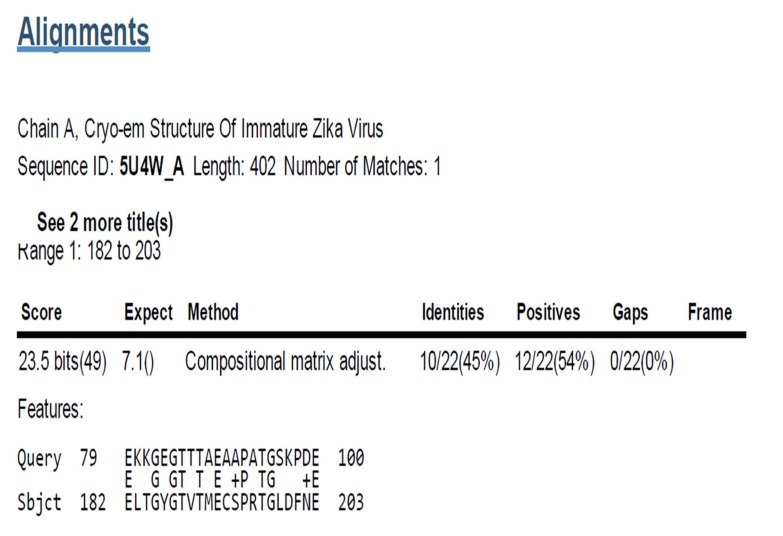
Neuromodulin similarity region in Chain A, Cryo-em Structure of Immature Zika V, Sequence ID: 5U4W_A Length: 402 Number of Matches: 1,Range 1: 182 to 203, Score: 23.5 bits (49), Expect: 7.1, Method: Compositional matrix adjust, Identities: 10/22(45%), Positives: 12/22(54%), Gaps: 0/22(0%).

**Figure 2:**
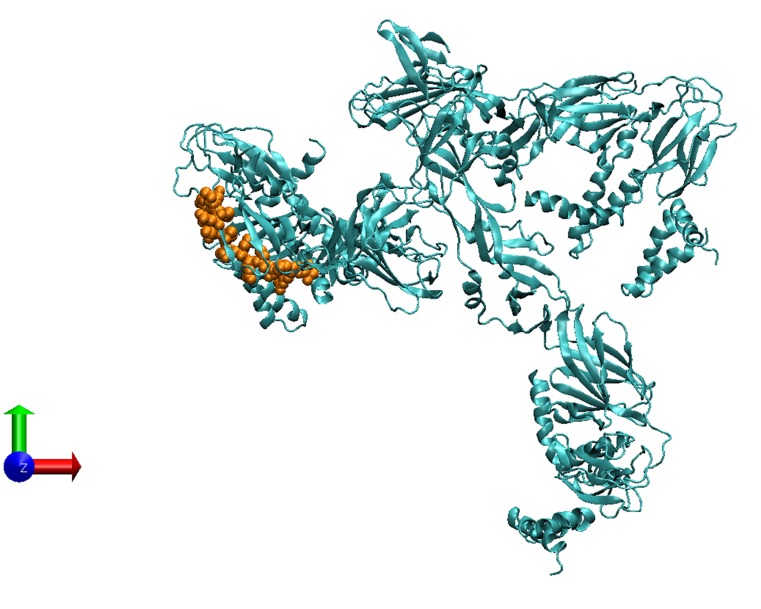
Neuromodulin similarity region in Chain A, Cryo-em Structure of Immature Zika V. The yellow chain of amino acids (ELTGYGTVTMECSPRT) is located on the ribbon model of 5U4W_A an output of VMD (Visual Molecular Dynamics) on the N-terminal side of the molecule.

Neuromodulin had a peptide sequence similarity to that of the present in the Cryo-em immature Zika V Protein Data Bank PDB. ID: 5U4W_A peptide sequence, which forms a part of polyprotein (Figure 1). The similarity of peptide region from 182 to 203 positions 'ELTGYGTVTMECSPRT' of Neuromodulin with Zika V protein has been superimposed and structurally modelled (Figure 2).

### Sequence similarity of Nestin to Zika V polyprotein

**Figure 3:**
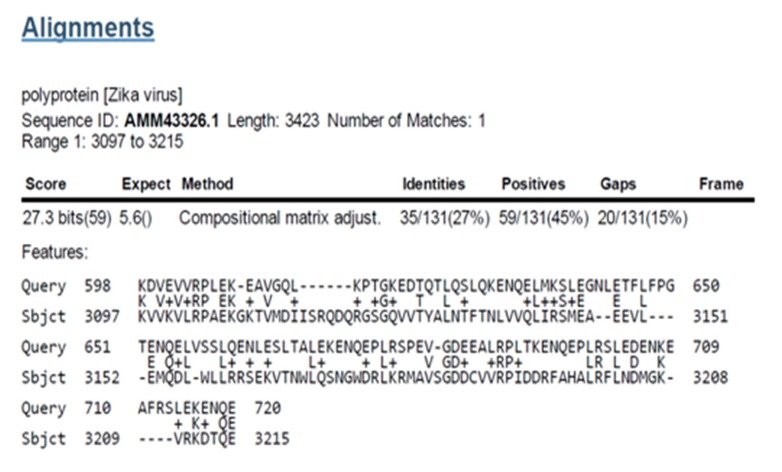
Nestin similarity region in polyprotein [Zika V], Sequence ID: AMM43326.1, Length: 3423, Number of Matches: 1, Range 1: 3097 to 3215, Score: 27.3 bits (59), Expect: 5.7, Method: Compositional matrix adjust, Identities: 35/131(27%), Positives: 59/131(45%), Gaps: 20/131(15%).

Nestin was identified to have sequence similarities to the Zika V polyprotein (Sequence ID: AMM43326.1) Amino acid range: 3097 to 3215 position by BLAST results (Figure 3).

### Sequence similarity of Bombesin to Zika V polyprotein

**Figure 4:**
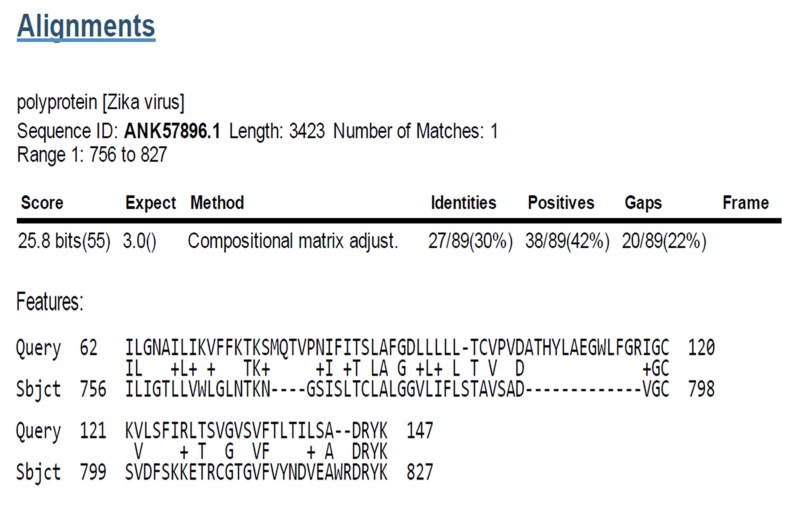
Bombesin similarity region in polyprotein [Zika V], Sequence ID: ANK57896.1, Length: 3423, Number of Matches: 1, Range 1: 756 to 827. Score: 25.8 bits (55), Expect: 3.0, Method: Compositional matrix adjust, Identities: 27/89(30%), Positives: 38/89(42%), Gaps: 20/89(22%).

Bombesin was identified to have sequence similarities with polyprotein Zika V (Sequence ID: ANK57896.1) Amino acid range: 62 - 147 position (Figure 4).

### Sequence similarity of Galanin to Zika V polyprotein

**Figure 5:**
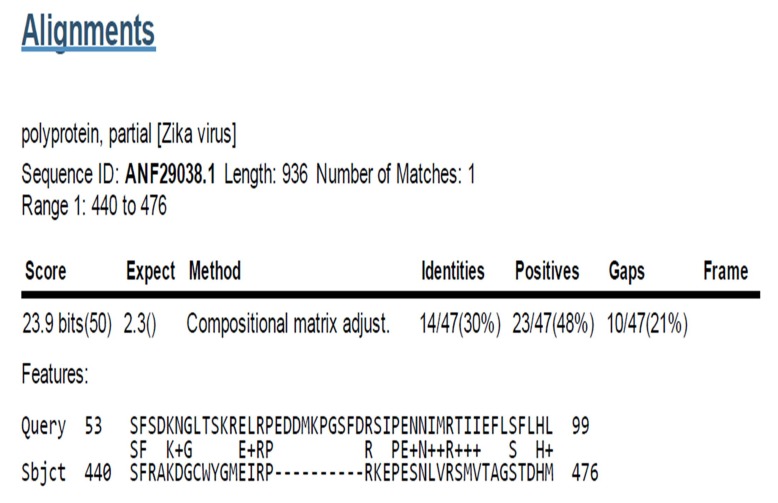
Galanin similarity region in polyprotein, partial [Zika V], Sequence ID: ANF29038.1, Length: 936, Number of Matches: 1, Range 1: 440 to 476. Score 23.9 bits (50), Expect 2.3, Method: Compositional matrix adjust, Identities: 14/47(30%), Positives: 23/47(48%), Gaps: 10/47(21%).

Galanin was identified to have similarities with the polyprotein partial Zika V (Sequence ID: ANF29038.1) Amino acid range: 440 - 476 position (Figure 5).

### Sequence similarity of Calcium-binding protein to Zika V: polyprotein

**Figure 6:**
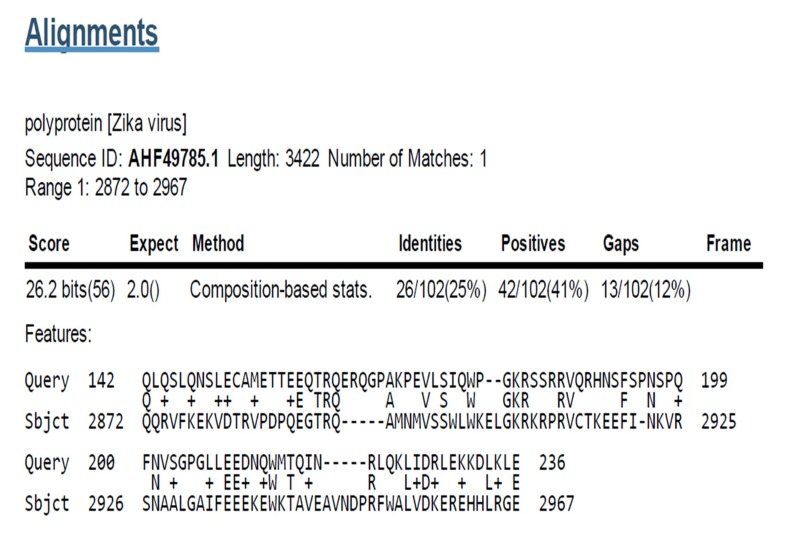
Calcium binding protein similarity region in polyprotein [Zika V], Sequence ID: AHF49785.1, Length: 3422, Number of Matches: 1, Range 1: 2872 to 2967Score: 26.2 bits (56), Expect: 2.0, Method: Composition-based stats, Identities: 26/102(25%), Positives: 42/102(41%), Gaps: 13/102(12%).

**Figure 7:**
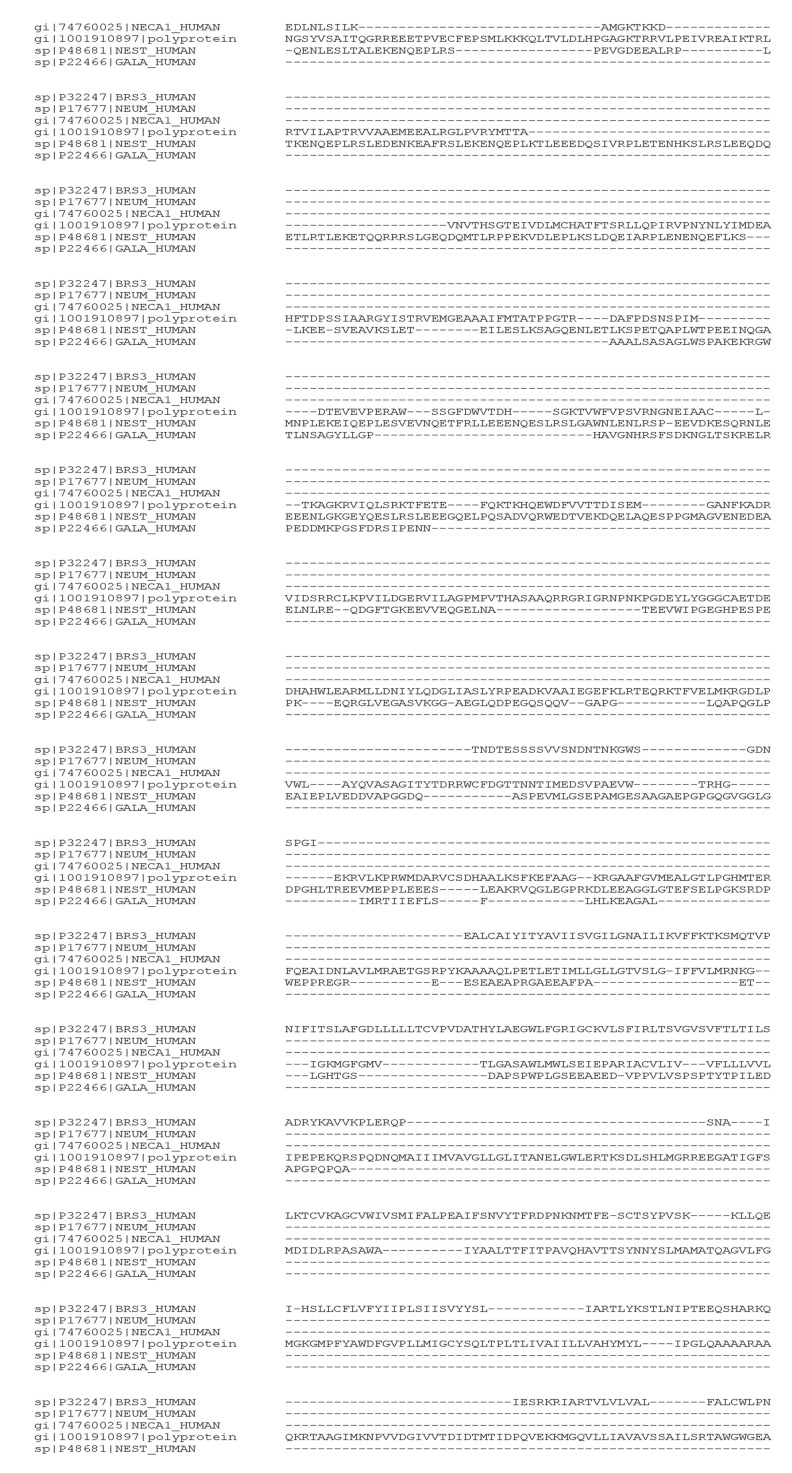
The segment of Zika virus polyprotein C (1659-2464 aa) that shows sequence similarities in multiple sequence alignment of human proteins. The sequences of polyprotein C were aligned with bombesin, neuromodulin, calcium binding protein, nestin and galanin using CLUSTAL O (1.2.4) for multiple sequence alignment.

Calcium Binding Proteins (CaBPs) were identified to have similarity with polyprotein Zika V (Sequence ID: AHF49785.1). Amino acid range: 2872 to 2967 position (Figure 6). Multiple sequence alignments were carried out for polyprotein C with Neuromodulin, Nestin, Bombesin, Galanin and Calcium-binding protein. Multiple sequence similarities were found in a broad region of amino acids 900 -3320 [23] (Figure 7). Comparative similarity percentages of Zika V polyprotein C with human proteins are shown in Table 2.

## Discussion

Bioinformatics is an exciting; exploratory method for peptide discovery towards the development of antimicrobial therapies and vaccination strategies [24]. The approach to identifying the similarities between host cell-viral proteins has now become facile with the extensive genomic and protein databases that exist [25]. The present study selected 63 human nerve proteins of which peptides of the Neuromodulin, Nestin, Bombesin, Galanin and Calcium-binding protein were found to have mimics with the Zika V proteins. The study discovered multiple similarity regions in polyprotein C of Zika V. This approach was different from the earlier published method, which selected pentapeptide epitopes in the human proteome database and BLAST against the Zika V proteome sequence. A vast number of pentapeptide matching/mimics was observed which were putative epitopes for autoimmunity. Our data strengthened the hypothesis of host autoimmunity due to the sequence and structural mimics of the Zika V with host peptides larger than pentapeptide [19]. In addition to causing autoimmunity in the host, the similarities could also have an influence on other metabolic pathways of the host cell. Human nerve protein Neuromodulin is a component of the motile growth cones. It is a membrane protein whose expression is widely correlated with nerve growth (axon elongation and effective regeneration response) [26]. Although the biological role of Neuromodulin is undetermined, the Nterminal region contains a calmodulin binding domain, sites for fatty acylation, membrane attachment and a protein kinase C phosphorylation site (Uniprot Data). A structural prediction of the C-terminal region suggests similarities to the side arms of neurofilaments, which could ultimately have a role in the formation of a dynamic membrane-cytoskeleton-calmodulin complex [27]. The sequence similarities identified in Zika V and Neuromodulin could alter membrane signal transduction and function of neurofilaments in the neuron, influence viral replication and further impair the immune surveillance system.

Nestin an intermediate filament protein is a stem cell marker expressed in the development of the central nervous system [28]. Nestin's similarity with polyprotein C of the Zika V could play a role in the pathogenesis of Zika V in the fetal brain. The similarity of Nestin with RNA-directed RNA polymerase (NS5) protein of the Zika V could influence host-pathogen interactions specifically encouraging viral proteome replication. It could also prevent the establishment of the cellular antiviral state by blocking the interferon-alpha/beta (IFN-alpha/beta) signalling pathway, inhibiting host TYK2 and STAT2 phosphorylation; thereby preventing activation of the JAK-STAT signalling pathway and to immune evasion [28].

Galanin is a peptide, which functions as a hormone that regulates the neuromodulation in the central and peripheral nervous systems. It is localised in neurosecretory granules and it could also function as a neurotransmitter. It has been shown to coexist with other peptide and amine neurotransmitters within individual neurons [30, 31]. The Galanin that shows similarity with the Zika V proteome is Envelope protein E. This protein is responsible for binding to host cell surface receptors and mediates fusion between viral and cellular membranes. Galanin peptides are associated with depression in Alzheimer's and the similarities of Galanin to Zika V polyprotein could be Zika V associated depression [32, 33].

Bombesin-like peptides are a large family, which are localised in CNS. In Xenopus laevis, the highest number of Bombesin binding sites was present in the brain and has a regulatory role in energy metabolism [29]. The similarity of the Zika V polyprotein with Bombesin could influence the energy metabolism of the fetal brain.

Calcium Binding Proteins (CaBPs are related to Calmodulins) are localised in the brain and sensory organs. They are an important components of Ca (2+) mediated cellular signal transduction, excitation-contraction coupling in muscle, neurotransmitter and hormone release and Ca2+-dependent gene transcription Calcium is the key element of adequate neuronal function in the body. The CaBP family regulates effectors such as voltage-gated Ca2+ channels in a Ca2+-dependent manner [34, 35, 36]. The similarity of the Zika V polyprotein with CaBP could have an interaction affecting the neuronal cell function.

All of the five nerve proteins Neuromodulin, Nestin, Galanin, Bombesin, Calcium binding protein had similarities to the Polyprotein C (3423 aa length) of the Zika V. Polyproteins [http://www.uniprot.org/ uniprot/Q32ZE] are a subgroup of non-structural major viral proteins (NSP) which are highly significant (prM, RNA-directed RNA polymerase NS5, NSP, 2A, 2B, 4A, 4B, Serine protease NS3, Peptide 2kPCBPs) in virus budding by attachment to the host cell membrane, gathering viral RNA into a nucleo-capsid to form the core of a mature virus particle within the host. During viral entry into the cell, the polyprotein induces genome penetration in host cytoplasm and migration into host cell nucleus where it modulates host functions [37]. The similarities identified in Nestin, Bombesin, Galanin, Calcium Binding Protein and Neuromodulin to their counterpart polyprotein C in ZIKV could help us identify peptide sequences which can regulate host cell [38].

Alternation from the normal cell state could cause biochemical and physiological changes in host signalling, transmission, metabolic alteration, inflammation, autoantibody (autoimmunity) and neuropathy. Increased rates of Guillain-Barre [39, 40] an aberrant physiological function affecting cardiac rhythm [41] have been associated with Zika V infection. The in silico search is the beginning of identifying host-pathogenic mimics.

The functionally relevant step after this is to publish the wet experimental data of confirmed mimics. These similarity overlapping regions will be interesting to analyse by wet experimentation in cell culture and animal experimental models to better understand the mechanism of host-pathogen interaction and to identify potential targets for drug and vaccine discovery.

## Conclusion

This paper identified Zika viral Polyprotein C (virulent factor) sequence similarities to Human proteins Neuromodulin, Nestin, Bombesin, Galanin, Calcium-binding proteins all of which are significant in host functions in the nervous tissue. Multiple sequence alignment identified a distinct region of the polyprotein C (959-1659 aa which encompasses Non-structural protein 1, 2A, 2B and Serine protease NS3) having identities to all the five human proteins of this study. This region has critical functions involved in immune evasion, pathogenesis and viral replication. In summary, the identified regions of human nerve proteins and the Zika Viral polyprotein C warrants further experimentation on their role in the pathogenesis.

## Competing interests

The authors declare that they have no competing interests.

## Authors’ contributions

PM and LS conceived the present study, design, interpretation of data and preparation of the manuscript. JL, LV, PDS, SS and RS were involved in interpretation and preparation of the manuscript.

## Figures and Tables

**Table 1:** List of nerve proteins used in the analysis

S. No	Proteins	Protein Code
1	Agrin	AGRN, O00468
2	Calbindin	CALB1,P05937
3	N-chimaerin	CHIN,P15882
4	Secretogranin-2	SCG2,P15882
5	Neuromodulin	NEUM,P13521
6	Kinesin	KIFC1,P17677
7	Tau protein	TAU,P10636
8	2',3'-cyclic-nucleotide 3'-phosphodiesterase	CN37,P09543
9	Myelin-associated glycoprotein	MAG,P20916
10	Myelin protein P0	MYP0,P25189
11	Myelin P2 protein	MYP2,P02689
12	Oligodendrocyte-myelin glycoprotein	OMGP,P23515
13	Brain-derived neurotrophic factor	BDNF,P23560
14	Ciliary neurotrophic factor	CNTF,P26441
15	Neurotrophin-3	NTF3,P20783
16	Beta-nerve growth factor	NGF,P01138
17	Nestin	NEST,P48681
18	Neurofilament heavy polypeptide	NFH,P12036
19	Neurogranin	NEUG,Q92686
20	Voltage-dependent T-type calcium channel subunit alpha-1G	CAC1G,O43497
21	Hippocalcin	HPCL1,P37235
22	Neurocalcin-delta	NCALD,P61601
23	Recoverin	RECO,P35243
24	Bombesin receptor subtype-3	BRS3,P32247
25	Kininogen-1/Bradykinin	KNG1,P01042
26	Calcitonin	CALC,P01258
27	Cholecystokinin	CCKN,P06307
28	Galanin peptides	GALA,P22466
29	Pro-neuropeptide Y	NPY,P01303
30	Neurotensin/neuromedin N	NEUT,P30990
31	Protein S100-B	S100B,P04271
32	Synapsin-1	SYN1,P17600
33	Probable tubulin polyglutamylase	TTLL1,O95922
34	Myelin basic protein	MBP,P02686
35	Protein phosphatase 1 regulatory subunit 1B	PPR1B,Q9UD71
36	Arf-GAP with GTPase, ANK repeat and PH domain-containing protein 2	AGAP2,Q99490
37	Cathepsin L2	CATL2,O60911
38	D(1A) dopamine receptor	DRD1,P21728
39	BDNF/NT-3 growth factors receptor	NTRK2,Q16620
40	Melanoma-associated antigen E1	MAGE1,Q9HCI5
41	Microtubule-associated protein 6	MAP6,Q96JE9
42	Protocadherin alpha-12	PCDAC,Q9UN75
43	Carboxypeptidase E	CBPE,P16870
44	Down syndrome cell adhesion molecule	DSCAM,O60469
45	Dyslexia-associated protein KIAA0319	K0319,Q5VV43
46	Uncharacterized protein KIAA1211-like	K121L,Q6NV74
47	Microtubule-associated protein 1B	MAP1B,P46821
48	Neuronal calcium sensor 1	NCS1,P62166
49	Neurofilament light polypeptide	NFL,P07196
50	Receptor expression-enhancing protein 2	REEP2,Q9BRK0
51	Secretogranin-3	SCG3,Q8WXD2
52	Ubiquitin carboxyl-terminal hydrolase isozyme L1	UCHL,P09936
53	Galactosylgalactosylxylosylprotein 3-beta-glucuronosyltransferase 1	B3GA1,Q9P2W7
54	Beta-1,4 N-acetylgalactosaminyltransferase 1	B4GN1,Q00973
55	Caprin-2	CAPR2,Q6IMN6
56	Dopamine beta-hydroxylase	DOPO,P09172
57	Protein FAM81A	FA81A,Q8TBF8
58	Mitogen-activated protein kinase 10	MK10,P53779
59	N-terminal EF-hand calcium-binding protein 1	NECA1,Q8N987
60	Neuroligin-3	NLGN3,Q9NZ94
61	Protein kinase C and casein kinase substrate in neurons protein 1	PACN1,Q9BY11
62	Sodium channel protein type 7 subunit alpha	SCN7A,Q01118
63	Clathrin coat assembly protein AP180	AP180,O60641

**Table 2:** Comparative similarity percentages of Zika V polyprotein C with human proteins.

S. No	Human Protein	Viral Protein	Sequence ID Zika V Polyprotein	Similarity Region Zika V Polyprotein	% Similarity
1	Neuro-modulin	Chain A, Cryoem Structure Of Immature Zika Virus	5U4W_A	182 to 203	54
2	Nestin	polyprotein	AMM43326.1	3097 to 3215	45
3	Bombesin	polyprotein	ANK57896.1	756 to 827	42
4	Galanin	Polyprotein, partial	ANF29038.1	440 to 476	48
5	Calcium binding protein	polyprotein	AHF49785.1	2872 to 2967	41
